# Etiology and Risk Factors of Acute Gastroenteritis in a Taipei Emergency Department: Clinical Features for Bacterial Gastroenteritis

**DOI:** 10.2188/jea.JE20150061

**Published:** 2016-04-05

**Authors:** Chao-Chih Lai, Dar-Der Ji, Fang-Tzy Wu, Jung-Jung Mu, Ji-Rong Yang, Donald Dah-Shyong Jiang, Wen-Yun Lin, Wei-Ting Chen, Muh-Yong Yen, Ho-Sheng Wu, Tony Hsiu-Hsi Chen

**Affiliations:** 1 Emergency Department, Taipei City Hospital, Ren-Ai Branch, Taipei, Taiwan; 2 Graduate Institute of Epidemiology and Preventive Medicine, College of Public Health, National Taiwan University, Division Biostatistics, Taipei, Taiwan; 3 Research and Diagnostic Center, Centers for Disease Control, Department of Health, Taipei, Taiwan; 4 Field Epidemiology Training Program, Centers for Disease Control, Taipei, Taiwan; 5 Infectious Disease Section, Taipei City Hospital, Taipei, Taiwan; 6 Department of Tropical Medicine, National Yang-Ming University, Taipei, Taiwan

**Keywords:** acute gastroenteritis, fecal leukocytes, fecal occult blood, norovirus, salmonella, *Giardia lamblia*, receiver operating characteristic, C-reactive protein

## Abstract

**Background:**

The causative pathogen is rarely identified in the emergency department (ED), since the results of cultures are usually unavailable. As a result, antimicrobial treatment may be overused. The aim of our study was to investigate the pathogens, risk factors of acute gastroenteritis, and predictors of acute bacterial gastroenteritis in the ED.

**Methods:**

We conducted a matched case-control study of 627 stool samples and 612 matched pairs.

**Results:**

Viruses (41.3%) were the leading cause of gastroenteritis, with noroviruses (32.2%) being the most prevalent, followed by bacteria (26.8%) and *Giardia lamblia* (12.4%). Taking antacids (adjusted odds ratio [aOR] 4.10; 95% confidence interval [CI], 2.57–6.53), household members/classmates with gastroenteritis (aOR 4.69; 95% CI, 2.76–7.96), attending a banquet (aOR 2.29; 95% CI, 1.64–3.20), dining out (aOR 1.70; 95% CI, 1.13–2.54), and eating raw oysters (aOR 3.10; 95% CI, 1.61–5.94) were highly associated with gastroenteritis. Elders (aOR 1.04; 05% CI, 1.02–1.05), those with CRP >10 mg/L (aOR 2.04; 95% CI, 1.15–3.62), or those who were positive for fecal leukocytes (aOR 2.04; 95% CI, 1.15–3.62) or fecal occult blood (aOR 1.97; 95% CI, 1.03–3.77) were more likely to be hospitalized in ED. In addition, presence of fecal leukocytes (time ratio [TR] 1.22; 95% CI, 1.06–1.41), abdominal pain (TR 1.20; 95% CI, 1.07–1.41), and frequency of vomiting (TR 0.79; 95% CI, 0.64–0.98) were significantly associated with the duration of acute gastroenteritis. Presence of fecal leukocytes (aOR 2.08; 95% CI, 1.42–3.05), winter season (aOR 0.45; 95% CI, 0.28–0.74), frequency of diarrhea (aOR 1.69; 95% CI, 1.01–2.83), and eating shrimp or crab (aOR 1.53; 95% CI, 1.05–2.23) were highly associated with bacterial gastroenteritis. The area under the receiver operating characteristic curve of the final model was 0.68 (95% CI, 0.55–0.63).

**Conclusions:**

Acute bacterial gastroenteritis was highly associated with season, frequency of diarrhea, frequency of vomiting, and eating shrimp or crab.

## INTRODUCTION

Acute gastroenteritis is a common disease in the emergency department (ED). Viruses are the leading cause of acute gastroenteritis presenting to the ED.^1^ Most cases of acute gastroenteritis are self-limiting, but some patients with more serious infection resulting from invasive bacterial and parasitic organisms may present with life-threatening dehydration and shock. The epidemiologic settings and clinical and laboratory features that are associated with acute gastroenteritis influence the probability of each pathogen.^2^ The causative pathogens are rarely identified in the ED because the results of cultures are usually unavailable due to time constraints or failure to obtain specimens.^3^ Nonetheless, antimicrobial treatment is still indispensable in patients with suspected invasive diarrhea, which is marked by the presence of fever, abdominal pain, fecal leukocytes, and hemoccult. However, clinical features of invasive diarrhea also present in cases of viral gastroenteritis, so antimicrobial treatment may be overused in the ED. Research on using clinical attributes to identify the cause of acute gastroenteritis in the ED is still limited. The aims of the present study were to investigate the pathogens and risk factors of acute gastroenteritis cases presenting to the ED, factors affecting the odds for admission, and the criteria for ancillary testing to diagnose bacterial gastroenteritis.

## METHODS

### Study design

A matched case-control study was conducted in in the ED of Ren-Ai branch of Taipei City Hospital. Data on 754 patients were collected from August 1, 2005, to July 31, 2009. Inclusion criteria for cases were: at least three loose stools or three instances of vomiting; or either diarrhea and/or vomiting plus two or more additional symptoms, including abdominal pain, fever, nausea, blood in the stool, or stool mucus. We excluded patients who were less than 15 years old; exhibited coughing, a sore throat, or runny nose; or were bedridden (defined as anyone who needs help to leave the bed).^1^ Each case patient was matched one-to-one with a non-gastroenteritis control patient of the same gender, age (within 5 years), and date of ED visit (within 1 month). If several control patients appeared for one case patient, the one admitted on the date nearest to the case’s ED visit date was selected. If the selected control refused to participate, we chose the non-gastroenteritis patient with the next nearest admission date to that of the case’s ED visit as the matched control. We followed this protocol until we found a matched control for each case.

We sent questionnaires to all participants after they gave consent to participate in the study. Socio-demographic information, clinical history of gastroenteritis, and factors responsible for the disease, such as consumption of food items, water, and beverages, dining location, travel history, contact with ill persons, contact with animals, habits, medications taken, and previous morbidity, were collected in the questionnaires (see eQuestionnaires). A case-case study was also used to determine the risk factors for admission, the duration of illness, and the predictor of bacterial gastroenteritis. Patients with bacterial gastroenteritis are defined as any positive results of stool culture or polymerase chain reaction (PCR) for bacteria, whether or not patients were positive for other pathogens.

We assumed that the estimated odds ratio was 1.29 (0.45/0.35), respectively. A two-sided McNemar test for no effect with a significance level of 0.05 was considered to indicate statistical significance. Hence, a sample size of 626 matched pairs was required.

### Specimen collection and laboratory methods

A total of 627 stool samples were collected immediately after visiting the ED or within 3 days after discharge. Follow-up telephone interviews with all participants were conducted 7–10 days after the ED visit. Blood cultures were performed for patients with fevers. All specimens were sent to the Centers for Disease Control, Taiwan and were analyzed for viruses, parasites, and bacteria.^1^ In this study, the newly developed PCR assays for detecting *B. fragilis* and *C. difficile* were performed.^4^

All blood tests (including blood cultures) and tests for fecal leukocytes and fecal occult blood were performed at the Taipei City Hospital laboratory.^1^ The threshold for positive fecal leukocytes was defined as more than 1 cell/high-power field (HPF), and fecal occult blood test was assessed using o-tolidine.

### Statistical analysis

Returned questionnaires were coded, and data were entered into Epi Info (version 3.43; U.S. Centers for Disease Control and Prevention, Atlanta, GA, USA) and analyzed using SAS software (release 9.3; SAS Institute Inc., Cary, NC, USA). Chi-square test or Fisher exact test were used for categorical data, and *t*-test or ANOVA were used for continuous data. Medians were compared using the Wilcoxon rank-sum test.

We estimated odds ratios (ORs) and 95% confidence intervals (CIs) using conditional logistic regression. Conditional logistic regression model with a stepwise selection procedure (*P* to enter <0.10; *P* to remove >0.05) was used to identify the most important determining factors for acute gastroenteritis.

In the case-case study, we estimated ORs and 95% CIs using the logistic regression model with a stepwise selection procedure (enter: *P* < 0.10; remove: *P* > 0.05) to identify the most important determining factors for admission and predictors of bacterial gastroenteritis. The duration of illness, from the onset of symptoms to the final resolution of symptoms, was evaluated using an accelerated failure time (AFT) model with a Weibull distribution. Parameter coefficients connected with the AFT model were reported as time ratio (TR; e^β^) in illness duration, a TR >1 is associated with a prolonged survival time, whereas a TR <1 is associated with a decrease in survival time. The AFT model, a parametric approach, can estimate the baseline hazard, which cannot be obtained with the Cox proportional hazards regression. Therefore, the AFT model is useful for analysis of time to event (the duration of illness). The AFT formulation allows the derivation of a time ratio, which is easier to interpret than the Cox proportional hazards regression model.^5^ In addition, the AFT model does not need the assumption of proportional hazards and provides more precise results in the analysis of censored data compared with the Cox proportional hazards regression model.

For comparison of the discriminatory capability of fecal leukocyte testing and the final model, a summary receiver operating characteristic (ROC) curve was constructed. A summary ROC curve was plotted to present the true-positive rate (sensitivity) against the false-positive rate (1 − specificity).^6^ Calculation of the area under the ROC curve (AUROC) provides a tool for comparison of the discriminatory capacity of different ancillary tests and risk factors.

### Ethics

The study was approved by the Taipei City Hospital Institutional Review Board.

## RESULTS

Of 2341 patients with gastroenteritis during the study period, only 754 (32.2%) patients participated in our study. Among the 754 patients who met the study criteria and returned completed and usable questionnaires, 627 stool samples were collected. There were no significant differences between participants and non-participants in terms of age, gender, and diarrhea symptoms. The participants’ characteristics and reported symptoms are shown in Table 1. Younger patients and patients with minor symptoms/signs (eg, less frequent diarrhea, less bloody stool, and stool with mucus) tended to have difficulty collecting their stool samples. A total of 59 (7.8%) patients were admitted to the hospital. Antibiotics were given to 76 (10.1%) patients with severe gastroenteritis symptoms in the ED. Median illness duration was 3.0 days (range, 1–55 days). One (0.1%) patient with unknown etiology died during admission, and the duration of illness of this dead case was treated as the right censored data (absence of event before study ended) in the accelerated failure time model.

**Table 1.** Characteristics of participants with acute gastroenteritis

Characteristic	Participants with stool test (*n* = 627)	Participants without stool test (*n* = 127)	*P*^a^
Age, years			
Median (range)	36 (15–94)	29 (15–88)	<0.001
Gender, number (%)			
Male	286 (45.6)	50 (39.4)	0.239
Female	341 (54.4)	77 (60.6)	
Maximum frequency of diarrhea in one day			
Median	6	5	0.002
Range	1–50	1–42	
Maximum frequency of vomiting in one day			
Median	3	3	0.401
Range	1–20	1–10	
Symptom distribution, number (%)			
Diarrhea	533 (85.0)	96 (75.6)	0.017
Abdominal pain	366 (58.4)	65 (51.2)	0.135
Weakness	297 (47.4)	63 (49.6)	0.654
Vomiting	256 (40.8)	55 (43.3)	0.605
Nausea	235 (37.5)	52 (40.9)	0.463
Abdominal bloating	206 (32.9)	27 (21.3)	0.010
Poor appetite	187 (29.8)	30 (23.6)	0.159
Myalgia	181 (28.9)	33 (26.0)	0.511
Fever	113 (18.0)	19 (15.0)	0.408
Tenesmus	45 (7.20)	13 (10.2)	0.238
Blood in stool	31 (4.90)	1 (0.80)	0.029
Mucus in stool	126 (20.1)	15 (11.8)	0.029

^a^Chi-square test was used to calculate *P* values for gender and symptom distribution. Wilcoxon rank sum test was used to calculate *P* value for age, maximum daily diarrheic stool frequency, and maximum daily vomiting frequency. Fisher exact test was used to calculate *P* values for “Blood in stool”.

Stool specimens were obtained immediately in 202 patients, and the others were obtained within 24 hours. The mean duration of taking stool specimens (ie, the time between arriving in the ED and obtaining stool specimens) did not differ significantly between the discharge group (mean 15.2 hours) and the admission group (mean 15.3 hours). There was also no difference in the proportion of patients with pathogen findings between the discharge (63.3%) and the admission (63.2%) groups.

Distributions of microbiologic findings are shown in Table 2. Viruses (41.4%) were identified as the leading cause of gastroenteritis, with norovirus (32.3%) being the most prevalent. Patients with higher diarrhea frequency (≥6 times per day) were more likely to have detected pathogens (68.1% vs 59.5%; *P* = 0.026). Of the 152 patients with blood cultures, 6 (3.9%) were positive for *Bacteroides fragilis*, *E. coli*, or *Aeromonas salmonicida*. The distributions of fecal occult blood and pus cells among different etiologies are listed in Table 2. A high co-infection rate (15.3%) was noted in this study. Only 26 (16.4%) cases with bacterial gastroenteritis received empirical antibiotics treatment.

**Table 2.** Microbiologic findings among participants

	Total (*n* = 627)	Positive fecal leukocyte (*n* = 199)	Positive fecal hemoccult (*n* = 258)	Both positive^a^ (*n* = 143)
	Number (%)	Number (%)	Number (%)	Number (%)
**Viral pathogens**	259 (41.3)	67 (33.7)	92 (35.7)	42 (29.4)
Norovirus	202 (32.2)	49 (24.6)	69 (26.7)	31 (21.7)
Rotavirus	46 (7.30)	15 (7.50)	18 (7.00)	8 (5.60)
Astrovirus	8 (1.30)	3 (1.50)	4 (1.60)	3 (2.10)
Sapovirus	7 (1.10)	1 (0.50)	2 (0.80)	1 (0.70)
Adenovirus	2 (0.30)	0 (0.00)	1 (0.40)	0 (0.00)
**Bacterial pathogens**	168 (26.8)	76 (38.2)	91 (35.3)	62 (43.4)
*Shigella spp.*	3 (0.50)	3 (1.50)	3 (1.20)	3 (2.10)
*Salmonella spp.*	29 (4.60)	16 (8.00)	19 (7.40)	14 (9.80)
*Vibrio parahaemolyticus*	34 (5.40)	16 (8.00)	24 (9.30)	14 (9.80)
*Aeromonas spp.*	5 (0.80)	4 (2.00)	4 (1.60)	4 (2.80)
*Campylobacter spp.*	12 (1.90)	7 (3.50)	8 (3.10)	6 (4.20)
*Plesiomonas shigelloides*	2 (0.30)	1 (0.50)	2 (0.80)	1 (0.70)
*Staphylococcus* with related enterotoxin	18 (2.90)	5 (2.50)	7 (2.70)	3 (2.10)
sDEC	62 (9.90)	22 (11.1)	22 (8.50)	15 (10.5)
*Clostridium difficile*	14 (2.20)	9 (4.50)	9 (3.50)	8 (5.60)
Toxigenic Bacteroides *fragilis*	8 (1.30)	3 (1.50)	4 (1.60)	2 (1.40)
**Pathogenic parasites**	78 (12.4)	19 (9.50)	29 (11.2)	13 (9.10)
*Giardia lamblia*	78 (12.4)	19 (9.50)	29 (11.2)	13 (9.10)
**Unknown pathogens**	230 (36.7)	73 (36.7)	87 (33.7)	49 (34.3)

sDEC, Suspected Diarrheagenic *E. coli*.

^a^Fecal leukocyte and fecal hemoccult are both positive.

### Matched case-control study

Our case-control study sample consisted of 612 matched pairs (267 males and 345 females). Factors significantly associated with gastroenteritis in univariate analysis are shown in Table 3. According to the final multivariate conditional logistic regression analysis, five variables were found to have statistically significant associations with the gastroenteritis cases: taking antacids (adjusted OR 4.10; 95% CI, 2.57–6.53), having household members/classmates with gastroenteritis (adjusted OR 4.69; 95% CI, 2.76–7.96), attending a banquet (adjusted OR 2.29; 95% CI, 1.64–3.20), dining out (adjusted OR 1.70; 95% CI, 1.13–2.54), and eating raw oysters (adjusted OR 3.10; 95% CI, 1.61–5.94).

**Table 3.** Risk factors for gastroenteritis in the matched case-control study (612 paired participants)

Exposure	Univariate analysis		Multivariate model^b^	
	
	OR	95% CI	Adjusted OR	95% CI
Taking antacids within 1 month prior to illness	3.38	(2.34, 4.88)	4.10	(2.57, 6.53)
Household members/classmates with gastroenteritis^a^	4.64	(2.92, 7.35)	4.69	(2.76, 7.96)
Attending a banquet^a^	2.50	(1.91, 3.27)	2.29	(1.64, 3.20)
Dining out^a^	2.32	(1.68, 3.20)	1.70	(1.13, 2.54)
Eating raw oysters^a^	2.82	(1.62, 4.91)	3.10	(1.61, 5.94)
Eating honey peaches^a^	1.91	(1.14, 3.20)	—	—
Drinking bottled water^a^	1.66	(1.30, 2.12)	—	—
Eating shrimp/crab^a^	1.45	(1.16, 1.83)	—	—
Attending open-air banquet^a^	5.33	(1.55, 18.30)	—	—
Eating at a Chinese/Western restaurant^a^	2.45	(1.83, 3.28)	—	—
Eating at street catering^a^	2.50	(1.35, 4.65)	—	—
Eating at a noodle shop^a^	1.35	(1.03, 1.76)	—	—
Eating raw fish^a^	1.57	(1.16, 2.13)	—	—
Eating clam/shells (other than raw oysters)^a^	1.54	(1.21, 1.96)	—	—
Changing a diaper^a^	1.73	(1.11, 2.72)	—	—
Eating a cold side dish^a^	1.27	(0.99, 1.63)	—	—
Eating salad^a^	1.19	(0.94, 1.50)	—	—
Eating pork^a^	1.17	(0.87, 1.58)	—	—
Eating beef^a^	1.14	(0.89, 1.44)	—	—

CI, confidence interval; OR, odds ratio.

^a^Exposed to these factors within 1 week prior to illness.

^b^Odds ratio and 95% confidence interval were derived from conditional logistic regression after stepwise selection.

### Case-case study

Our case-case study sample consisted of 627 cases. Significant differences were observed in mean illness duration (*P* = 0.006), C-reactive protein (CRP) level (*P* = 0.005), presence of fecal leukocyte (*P* < 0.001) and fecal occult blood (*P* < 0.001), and the number of cases with vomiting (*P* = 0.036) among cases of acute gastroenteritis with different etiologies (Appendix eTable 1).

Gastroenteritis patients who were hospitalized were compared to those without hospital admission. The risk factors for hospitalization are shown in Table 4. In the final multivariable logistic regression model, age (adjusted OR 1.04; 95% CI, 1.02–1.05), CRP level >10 mg/L (adjusted OR 2.04; 95% CI, 1.15–3.62), and presence of fecal leukocyte (adjusted OR 2.04; 95% CI, 1.08–3.86) and fecal occult blood (adjusted OR 1.97; 95% CI, 1.03–3.77) were found to have statistically significantly associations with hospital admission.

**Table 4.** Risk factors for admission of patients with acute gastroenteritis to emergency department

Characteristic	Univariable analysis		Multivariable analysis	
	
	OR	(95% CI)	OR	(95% CI)
Age	1.04	(1.02, 1.05)	1.04	(1.02, 1.05)
Male gender	0.79	(0.45, 1.37)	—	—
WBC >10^4^ count/µL	1.51	(0.87, 2.65)	—	—
CRP >10 mg/L	2.05	(1.18, 3.56)	2.04	(1.15, 3.62)
Pathogens				
Bacteria	2.21	(1.27, 3.87)	—	—
Non-bacteria	1.00			
Fecal pus cell	3.09	(1.78, 5.37)	2.04	(1.08, 3.86)
Fecal occult blood	2.69	(1.53, 4.72)	1.97	(1.03, 3.77)
Fever	1.55	(0.82, 2.94)	—	—
Abdomen pain	1.76	(0.98, 3.18)		
Frequency of vomiting >5 times/day	0.74	(0.28, 1.91)	—	—
Frequency of diarrhea >10 times/day	0.92	(0.40, 2.11)	—	—
Household members with gastroenteritis	1.27	(0.65, 2.50)	—	—
Taking antacids within 1 month prior to illness	1.61	(0.88, 2.94)	—	—
Eating shrimp/crab	0.62	(0.35, 1.08)	—	—
Eating raw oysters	0.95	(0.28, 3.22)	—	—

CI, confidence interval; OR, odds ratio.

Factors affecting duration of illness of patients with acute gastroenteritis are shown in Table 5. In the univariate analysis of the AFT model, presence of fecal leukocytes (TR 1.22; 95% CI, 1.06–1.41), abdominal pain (TR 1.23; 95% CI, 1.07–1.41), and frequency of vomiting (>5 times/day; TR 0.79; 95% CI, 0.64–0.98) were significantly associated with the duration of illness. Presence of fecal leukocytes (TR 1.19; 95% CI, 1.06–1.41), abdominal pain (TR 1.20; 95% CI, 1.05–1.38), and frequency of vomiting (TR 0.79; 95% CI, 0.64–0.97) remained statistically significant in the multivariable model.

**Table 5.** Results from accelerated failure time model for illness duration of patients with acute gastroenteritis

Characteristic	Univariable analysis		Multivariable analysis	
	
	Time ratio in illness duration (95% CI)^#^	*P* value	Time ratio in illness duration (95% CI)^#^	*P* value
Age	1.00 (1.00, 1.01)	0.497	—	—
Male gender	0.98 (0.84, 1.16)	0.846	—	—
Bacterial pathogens	1.07 (0.89, 1.28)	0.460	—	—
Fecal leukocyte	1.22 (1.06, 1.41)	0.006	1.19 (1.06, 1.41)	0.014
Frequency of vomiting >5 times/day	0.79 (0.64, 0.98)	0.028	0.79 (0.64, 0.97)	0.025
Frequency of diarrhea >10 times/day	0.94 (0.77, 1.14)	0.525		
Abdominal pain	1.23 (1.07, 1.41)	0.003	1.20 (1.05, 1.38)	0.007
Fever	1.01 (0.85, 1.20)	0.950		
Fecal occult blood	0.97 (0.84, 1.11)	0.624		

CI, confidence interval.

^#^The corresponding regression coefficients in the above models can be obtained by taking the logarithm of time ratio.

Factors significantly associated with bacterial gastroenteritis based on univariate analysis are presented in Table 6. According to our results from the final multivariable logistic analysis, four variables were found to have statistically significant associations with the bacterial gastroenteritis cases: presence of fecal leukocytes (adjusted OR 2.08; 95% CI, 1.42–3.05), winter (adjusted OR 0.45; 95% CI, 0.28–0.78), frequency of diarrhea >10 times/day (adjusted OR 1.69; 95% CI, 1.01–2.83), and eating shrimp or crab (adjusted OR 1.53; 95% CI, 1.05–2.23).

**Table 6.** Risk factors for bacterial gastroenteritis comparing with non-bacterial gastroenteritis

Factors	Univariable analysis		Multivariable analysis	
	
	OR	95% CI	Adjusted OR	95% CI
Age	1.01	(1.00, 1.01)	—	—
Male gender	0.93	(0.65, 1.33)	—	—
WBC >10^4^ count/µL	1.35	(0.94, 1.93)	—	—
CRP >10 mg/L	1.45	(1.01, 2.07)	—	—
Fecal leukocyte	2.19	(1.52, 3.17)	2.08	(1.42, 3.05)
Fecal occult blood	1.96	(1.37, 2.81)	—	—
Abdomen pain	1.10	(0.76, 1.57)		
Fever	1.40	(0.90, 2.17)	—	—
Shock (systolic blood pressure <80 mm Hg)	1.08	(0.38, 3.08)	—	—
Frequency of vomiting >5 times/day	0.48	(0.25, 0.93)	0.54	(0.27, 1.08)
Frequency of diarrhea >10 times/day	1.75	(1.07, 2.86)	1.69	(1.01, 2.83)
Season Summer	1.51	(0.99, 2.32)	1.54	(0.99, 2.40)
Winter	0.39	(0.24, 0.63)	0.45	(0.28, 0.74)
Spring and Autumn	1.00	—	1.00	—
Eating shrimp/crab	1.46	(1.02, 2.09)	1.53	(1.05, 2.23)
Eating raw fish	1.53	(1.00, 2.36)		
Taking antacids within 1 month prior to illness	1.57	(0.76, 3.25)		
Household members with gastroenteritis	1.30	(0.83, 2.03)		
Eating raw oysters	1.19	(0.58, 2.47)		

CI, confidence interval; OR, odds ratio.

The sensitivity, specificity, and positive and negative predictive values of the presence of fecal leukocytes as a predictor of bacterial gastroenteritis were 44.9%, 72.9%, 37.2%, and 78.7%, respectively. The sensitivity, specificity, and positive and negative predictive values of the presence of fecal occult blood as a predictor of bacterial gastroenteritis were 53.3%, 63.2%, 34.1%, and 79.1%, respectively. Respective accuracy measures of CRP level (>10 mg/L) as a predictor of bacterial gastroenteritis were 48.5%, 60.6%, 30.5%, and 76.7%. The ROC curve for CRP level (>10 mg/L), presence of fecal occult blood, presence of fecal leukocytes, and the composite of factors included in our final model (presence of fecal leukocytes, frequency of diarrhea >10 times/day, frequency of vomiting >5 times/day, season, and eating shrimp or crab) is shown in Figure. The respective AUROCs were 0.55 (95% CI, 0.50–0.59), 0.58 (95% CI, 0.54–0.63), 0.59 (95% CI, 0.55–0.63) and 0.68 (95% CI, 0.55–0.63). The positive and negative predictive values of our final model were 60.0% and 75.4%, respectively.

**Figure.**
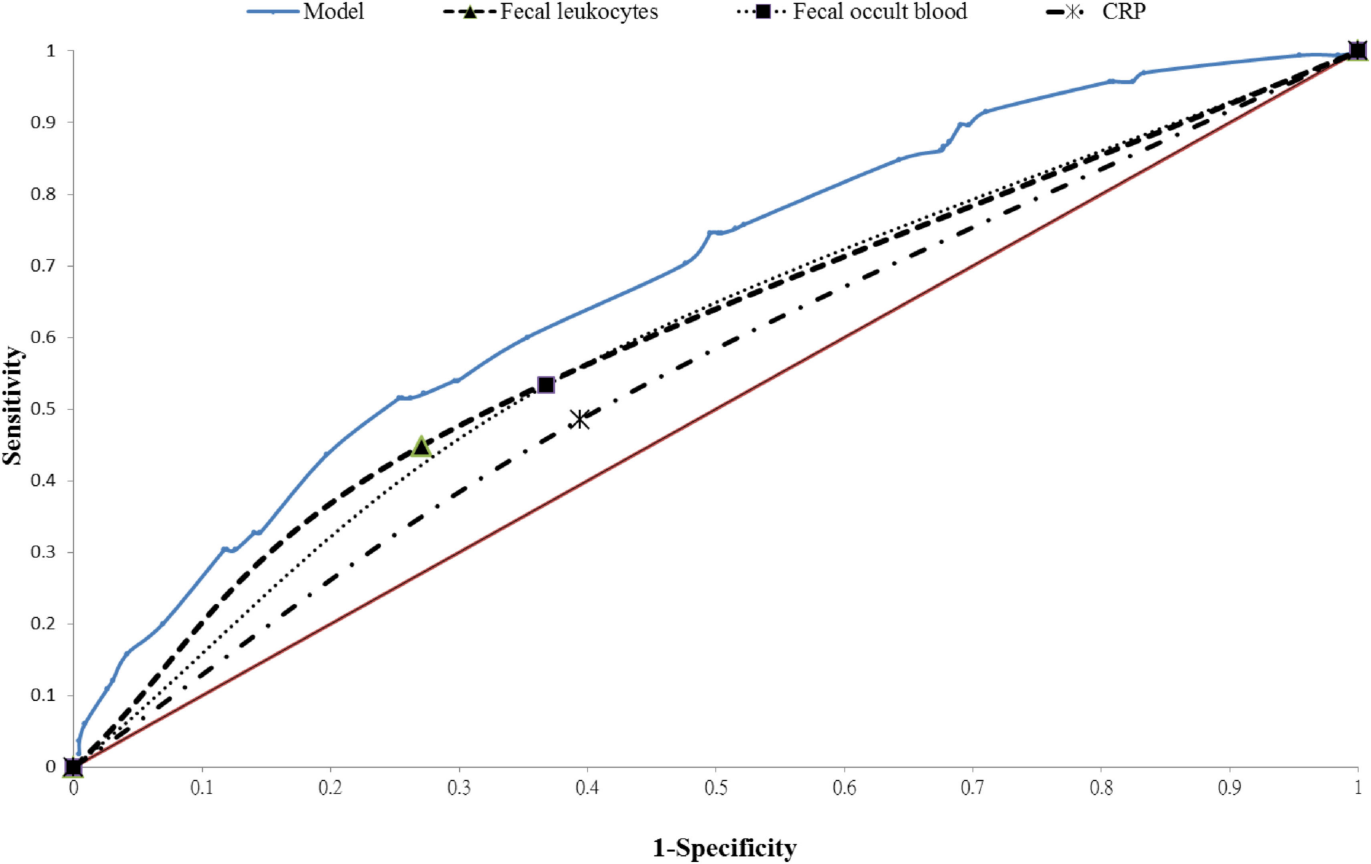
Receiver operating characteristic (ROC) curve for the ancillary test. The area under the ROC curve is 0.681 for our model, 0.589 for fecal leukocytes, 0.583 for fecal occult blood, and 0.546 for CRP (>10 mg/L).

## DISCUSSION

The current study assesses many aspects, including the etiology and risk factors of gastroenteritis in adolescents and adults, as well as factors affecting the odds for admission, the duration of illness, and ancillary testing for bacterial gastroenteritis in the ED. We found that viral infection was the leading cause of gastroenteritis and tends to cause gastroenteritis in winter. Older age, elevated CRP level, presence of fecal leukocytes, and presence of fecal occult blood were significantly associated with hospital admission. In addition, presence of fecal leukocytes, frequency of vomiting, and abdominal pain were significantly associated with the duration of illness. Presence of fecal leukocytes, winter, frequency of diarrhea >10 times/day, and eating shrimp or crab were highly associated with bacterial gastroenteritis. The presence of fecal leukocytes or fecal occult blood and elevated CRP level (>10 mg/L) have low sensitivity and specificity for predicting bacterial gastroenteritis. The AUROC of elevated CRP level, presence of fecal occult blood, presence of fecal leukocytes, and presence of all risk factors included in our final model were 0.546, 0.583, 0.589, and 0.681, respectively.

Our finding that norovirus was the leading cause of gastroenteritis cases in both adolescents and adults agrees with previous reports.^1^^,^^7^^–^^10^ *Clostridium difficile* was not thought to be a dominant pathogen for community-acquired diarrhea until Huhulescu et al proved it.^7^ However, community-acquired *Clostridium difficile* is still not a great problem in Taipei: it was identified in only 2.2% of all cases in our study. About 15.3% of patients were diagnosed with co-infection with bacteria and other non-bacterial pathogens; the relatively high detection rate of non-bacterial pathogens was due to our use of PCR to detect norovirus, giardia, *B. fragilis*, and *C. difficile*. The majority of co-infected patients were co-infected with norovirus and other pathogens. It should be noted that norovirus may not be the cause of illness in all cases that were positive for norovirus because healthy people can deliver false-positive results on PCR.^11^ We also observed a higher pathogen detection rate for patients with 6 or more daily episodes of diarrhea, which was similar to the results of a previous study.^12^

About 36.7% of gastroenteritis cases in our study were unexplained. Picobirnavirus may account for a proportion of these unknown cases, according to recent studies.^13^^,^^14^ Further studies are needed to clarify the possible pathogenicity of unexplained gastroenteritis.

Our findings about the risk factors of gastroenteritis are similar to previous studies. Dining out (defined as eating anywhere away from one’s home) was found to be associated with food poisoning cases at the ED in previous studies.^1^^,^^15^ Attending a banquet has also been associated with outbreaks of gastroenteritis.^1^^,^^16^ Decreasing gastric acidity is a risk factor for infectious diarrhea-related illnesses^1^^,^^17^ because gastric acid is capable of killing ingested bacteria.^18^ Household transmission is a known risk factor for gastroenteritis.^1^^,^^19^^–^^21^ Eating raw oysters has also been shown to increase the risk of gastroenteritis.^1^^,^^22^^–^^25^

Most gastroenteritis tends to be self-limiting and requires only supportive therapy. Antibiotic treatment is suggested in patients with a suspected invasive process and severe diarrhea, systemic symptoms, fever, or abdominal pain, as well as in patients who show toxic signs.^3^ However, fever and abdominal pain were not predictors of bacterial diarrhea in the present study. Further, eating shrimp or crab has been reported to be associated with diarrheagenic *E. coli*,^26^ *Vibrio* spp.,^27^^–^^29^ *Aeromonas* spp.,^30^ and *Listeria monocytogenes*^31^ infection in previous studies. Eating shrimp or crab was a predictor of bacterial gastroenteritis in the present study. Diarrhea in winter tends to be viral gastroenteritis because norovirus and rotavirus spread more easily in winter than in other seasons.

Though the presence of fecal leukocytes or fecal occult blood had low power for prediction of bacterial gastroenteritis, they were highly associated with hospital admission, and patients with fecal leukocytes were also found to have longer duration of illness than those without fecal leukocytes. However, patients with higher frequency of vomiting were found to have shorter duration of illness because such symptoms tended to result from viral gastroenteritis and no severe dehydration.

The sensitivity and specificity of microscopy for fecal leukocytes was heterogeneous in previous studies.^32^ No consistent trend in the test threshold (cells per HPF) to any of the diagnostic parameters has been observed. Our study demonstrated that the AUROC of bacterial gastroenteritis prediction was elevated after adding other predictors (winter, frequency of vomiting, frequency of diarrhea, and eating shrimp or crab) to the ED. The epidemiological evidence and clinical evidence were associated with the probability of “not norovirus” from as low as 8% to as high as 100%.^2^ However, there are still no ancillary tests or good models to predict bacterial gastroenteritis, and the guidelines for antimicrobial treatment of acute gastroenteritis are also ambiguous. One study reported that body temperature, abdominal pain, leukocyte count in stool, and poor dietary hygiene can be used as predictors of acute bacterial diarrhea in hospitalized patients.^33^ The AUROC was 0.975 (95% CI, 0.962–0.987) in their study. As acute bacterial diarrhea is also highly dependent on the environments in which patients live, the AUROC result may vary from study to study. The diagnostic accuracy of bacterial gastroenteritis using our model was not good enough due to the high percentage of cases with unknown etiology and co-infection with viruses in our study group. Further studies on the prediction of non-viral or bacterial gastroenteritis using the Bayesian approach should be considered.

The case-case comparison is more efficient and less biased than standard case-control studies for determining exposure to infectious agents, but general factors that are responsible for whether acute gastroenteritis occurs after the exposure cannot be studied.^34^ Selection bias may result from the low response rate of the ED cases. Greater frequency of diarrhea was noted in enrolled cases compared to in non-participators,^1^ and younger patients and patients with minor symptoms/signs were less likely to provide stool samples. In addition, patients with longer duration of gastroenteritis were more likely to visit the ED (ie, length bias) than those with shorter duration of illness. However, selection bias was minimized in this study by adjusting for these factors, including the frequency of diarrhea, age, and duration of gastroenteritis, when they were measured in all study subjects. Our findings tend to overestimate the positive detection rate of diarrheagenic *E. coli* using the O serotyping method alone.^35^

In summary, taking antacids, having household members/classmates with gastroenteritis, attending a banquet, dining out, and eating raw oysters were highly associated with gastroenteritis. The elderly and those with elevated CRP levels and the presence of fecal leukocytes and fecal occult blood tended to be hospitalized in the ED. The presence of fecal leukocytes, winter, frequent diarrhea, and eating shrimp or crab were highly associated with bacterial gastroenteritis.

## ONLINE ONLY MATERIALS

eTable 1. Characteristics of participants with acute gastroenteritis in different etiologic groups.

eQuestionnaire. Questionnaire of Gastroenteritis Investigation.
