# Supplementary material for: Etiology and Risk Factors of Acute Gastroenteritis in a Taipei Emergency Department: Clinical Features for Bacterial Gastroenteritis
Source: J Epidemiol. 2016 Apr 5;26(4):216–23. doi: 10.2188/jea.JE20150061 (PMC4808689; doi:10.2188/jea.JE20150061)
Supplement: eTable 1. [file je-26-216-s001.pdf]

eTable 1. Characteristics of participants with acute gastroenteritis in different etiologic groups

Factors	Pathogens						*p*
	Virus (n=174)	Bacteria (n=106)	Parasite (n=21)	Co-infection		Unknown (n=230)	
				Including bacteria (n=59)	Excluding bacteria (n=37)		
Maximum frequency of diarrhea in one day							
Mean (SD)	7.68 (9.44)	9.04 (6.03)	7.21 (2.92)	7.96 (5.07)	6.92 (4.63)	6.91 (5.39)	0.161
Maximum frequency of vomiting in one day							
Mean (SD)	4.39 (3.90)	3.76 (2.97)	3.43 (2.56)	3.53 (2.76)	4.30 (3.08)	3.89 (2.98)	0.750
Mean illness duration							
Mean (SD)	1.84 (2.31)	3.63 (3.72)	2.53 (2.48)	2.65 (3.36)	2.67 (2.04)	3.00 (5.09)	0.006[a]
WBC ($\times 10^3$ count/μL)							
Mean (SD)	10.83 (3.92)	11.41 (5.03)	9.95 (3.49)	12.10 (10.37)	10.21 (4.04)	10.80 (4.22)	0.355
CRP (mg/L)							
Mean (SD)	15.39 (16.71)	25.85 (32.28)	15.80 (20.05)	17.30 (18.68)	18.97(21.80)	15.48 (22.68)	0.005[b]
Positive fecal leukocyte	40 (25.3%)	52 (51.0%)	2 (9.5%)	22 (38.6%)	10 (27.8%)	84 (36.5%)	<0.001[c]
Positive fecal occult blood	62 (35.6%)	69 (65.1%)	5 (23.8%)	19 (32.2%)	16 (43.2%)	96 (26.9%)	<0.001[c]

SD, standard deviation.

[a] bacteria vs. virus group: F value=10.6, *p*=0.001 in mean illness duration; unknown vs virus group: F value=12.8, *p*<0.001 in mean illness duration;

[b] bacteria vs. virus group: F value=13.8, *p*<0.001 in CRP; F value=27.3 , *p*<0.001 in fecal occult blood;

[c] bacteria vs. virus group: chi-square=18.7, $p<0.001$ in fecal pus cell and chi-square=24.4, $p<0.001$ in fecal occult blood;

eTable 1. Characteristics of participants with acute gastroenteritis in different etiologic groups (continued)

Symptom distribution Number (%)	Pathogens						*p*
	Virus (n=174)	Bacteria (n=106)	Parasite (n=21)	Co-infection		Unknown (n=230)	
				Including bacteria (n=59)	Excluding bacteria (n=37)		
Diarrhea	151 (86.8)	92 (86.8)	19 (90.5)	46 (78.0)	31 (83.8)	194 (84.4)	0.6076
Abdominal pain	90 (51.7)	67 (63.2)	17 (81.0)	32 (54.2)	24 (64.9)	136 (59.1)	0.0841
Vomiting	88 (50.6)	33 (31.1)	7 (33.3)	24 (40.7)	10 (40.5)	89 (38.7)	0.0363[d]
Nausea	68 (39.1)	33 (31.1)	6 (28.6)	20 (33.9)	17 (46.0)	91 (39.6)	0.4659
Abdominal bloating	56 (32.2)	27 (25.5)	7 (33.3)	14 (23.7)	18 (48.7)	84 (36.5)	0.0630
Poor appetite	54 (31.0)	28 (26.4)	7 (33.3)	11 (18.6)	13 (35.1)	74 (32.2)	0.3620
Myalgia	61 (35.1)	33 (31.1)	4 (19.1)	17 (28.8)	13 (35.1)	86 (24.1)	0.1026
Fever	31 (17.8)	26 (24.5)	2 (9.52)	10 (17.0)	5 (13.5)	39 (17.0)	0.4407

[d] bacteria vs. virus group: chi-square =10.42, p=0.0012; unknown vs virus group: chi-square =5.87, p=0.015
