# Supplementary material for: Etiology and Risk Factors of Acute Gastroenteritis in a Taipei Emergency Department: Clinical Features for Bacterial Gastroenteritis
Source: J Epidemiol. 2016 Apr 5;26(4):216–23. doi: 10.2188/jea.JE20150061 (PMC4808689; doi:10.2188/jea.JE20150061)
Supplement: eQuestionnaire. [file je-26-216-s002.pdf]

e-Questionnaire. Questionnaire for Gastroenteritis Investigation

Interviewer: ______________________ Interview Date: ____ /___ /___

Hospital ID: ______________________

Basic Information

1. Name: _______________
2. Sex: □male □female
3. Birth date: _____ year _____ month _____ day
4. Education: □illiterate □grade 1-6 □grade 7-12 □grade 13-16
   □college/university □graduate
5. Are you a student? □yes □no
   If your answer is "yes," what is the name of your school?

____

6. What is your occupation? ____
7. Address: ____________ County/City: _____________Village/Town/District: _____________
8. How many rooms are there in your home? ________rooms
9. What is the size of your home? □<50 □50-80 □81-130 □131-180 □>180 m^2
10. When is the best available time to contact you after 1 week?
    □daytime (8:00 - 17:00) Phone Number: ________________
    □night time (17:00 - 21:00) Phone Number: ________________
    □either day or night (8:00 - 21:00) Phone Number: ________________

Medical Condition and Disease History

1. ED date of arrival/time: _____ year ____ month ____ day ____ hour ____ minute
2. Temperature: ________°C Pulse: _______ /min Breath: _________/min
3. Blood Pressure: SBP________ /DBP ________ mmHg
4. Do you have any of the following medical conditions? □hypertension □diabetes mellitus
   □AIDS □liver cirrhosis □kidney dialysis □pulmonary emphysema
   □chronic bronchitis □asthma □post-gastrectomy
   □taking steroids or immunosuppressants within the past month
   □receiving chemotherapy within the past month □none
5. Have you taken any of the following within one month prior to illness?
   □antibiotics □antacids □H_2 antagonists or proton pump inhibitors
   □colchicine □diet pills □Traditional Chinese medications □Chinese medicinal herbs
   □modern medicine □none
6. Have you had either diarrhea or vomiting symptoms in the past month? □yes □no
7. Have you previously been administered this questionnaire in the past month? □yes □no
8. Did you have any of the following symptoms/signs?

□nausea □vomiting □diarrhea □abdominal pain □abdominal distention
□anorexia □weakness □sore body □tenesmus □fever (>38 °C)
□chill □skin rash □headache □dizziness □convulsion
□cough □runny nose □sore throat □difficulty breathing/asthma
□others ____________
□none

(If you chose "none," please go to the next section – "Contact and Travel History")

4. When did you begin to have uncomfortable symptoms?
____ month ____ day, □morning □afternoon □night, ____ hour ____ minute
5. Which of these uncomfortable symptoms appeared first?
□vomiting □diarrhea □both
6. What is the maximum frequency of diarrhea per day? ______ times/day
7. What is the maximum frequency of vomiting per day? ______ times/day
8. When you experience diarrhea, what does your stool look like?
□watery □loose stool □solid (normal shape)
Was your stool accompanied by any of the following symptoms?
□ blood □ mucous □ none
9. Have you had hemorrhoids with bleeding before this attack? □yes □no
10. When did you have the symptoms indicated above?
Did you take any medicine without a doctor's prescription? □yes □no
Did you visit a doctor? □yes □no
If your answer is "yes," please give the name of the clinic/hospital:
____
If you visited a doctor, did the doctor take a stool sample? □yes □no

Contact and Travel History

1. With whom do you live? □family members □classmates □colleagues □friends
□alone □others (please specify) ____________________________________
If you live with family members, how many individuals are presently living with you? _____ adults _____ children
2. Have any of your family members, classmates, colleagues, friends, or neighbors had either vomiting or diarrhea symptoms over the past 4 weeks? □yes □no
If so, please specify the number of symptoms experienced for each individual
____
What was the frequency per day? __________
How long did the symptoms last? □<3 days □4-6 days □1-2 weeks
3. Have you had contact with any children over the past week? □yes □no
If your answer is "yes," do you have vomiting or diarrhea symptoms? □yes □no
4. Did you travel abroad last month?

□no

□yes - please specify the country: _______________,

When did you start the trip? ___ month ___ day

When did you return from the trip? ___ month ___ day

5. Did you travel locally in the last month?

□no

□yes - please specify the destination: _______________,

When did you start the trip? ___ month ___ day

When did you return from the trip? ___ month ___ day

Specific activities

1. Did you engage in any of following activities?

Exposure	Within 24 hours before symptoms	24-72 hours before symptoms	4-7 days before symptoms
Change a diaper	□Yes □No	□Yes □No	□Yes □No
Swimming	□Yes □No	□Yes □No	□Yes □No
Shake hands with others	□Yes □No	□Yes □No	□Yes □No
Take a bus	□Yes □No	□Yes □No	□Yes □No
Take an airplane	□Yes □No	□Yes □No	□Yes □No
Take mass rapid transit	□Yes □No	□Yes □No	□Yes □No
Touch pets or other animals	□Yes □No	□Yes □No	□Yes □No
Go to the theater/pub	□Yes □No	□Yes □No	□Yes □No
Wash hands before a meal	□Yes □No	□Yes □No	□Yes □No
Wash hands with soap	□Yes □No	□Yes □No	□Yes □No
Hugging or kissing	□Yes □No	□Yes □No	□Yes □No
Dine out	□Yes □No	□Yes □No	□Yes □No
Attend a banquet	□Yes □No	□Yes □No	□Yes □No
Attend an open-air banquet	□Yes □No	□Yes □No	□Yes □No

2. Did you experience any of the following in the week prior to the onset of symptoms?

See a fly while eating ☐Yes ☐No

See a cockroach while eating ☐Yes ☐No

Restaurant exposure

Did you eat at any of the following types of commercial food establishments?

Exposure	Within 1 week before symptoms	Exposure	Within 1 week before symptoms
Eat at a noodle shop	□Yes □No	Eat from a street truck	□Yes □No
Eat at a Japanese restaurant	□Yes □No	Eat at a hamburger fast food restaurant	□Yes □No
Eat at a Chinese/Western restaurant	□Yes □No		

Specific food exposure

1. Did you eat any dishes containing the following foods prior to the onset of symptoms?

Exposure	Within 24 hours before symptoms	24-72 hours before symptoms	4-7 days before symptoms
Eat salad	□Yes □No	□Yes □No	□Yes □No
Eat raw oysters	□Yes □No	□Yes □No	□Yes □No
Eat raw fish	□Yes □No	□Yes □No	□Yes □No
Eat raw eggs	□Yes □No	□Yes □No	□Yes □No
Eat over-easy eggs	□Yes □No	□Yes □No	□Yes □No
Eat clams/shellfish (other than oysters)	□Yes □No	□Yes □No	□Yes □No
Eat beef	□Yes □No	□Yes □No	□Yes □No
Eat pork	□Yes □No	□Yes □No	□Yes □No
Eat fish	□Yes □No	□Yes □No	□Yes □No
Eat chicken	□Yes □No	□Yes □No	□Yes □No
Eat mutton	□Yes □No	□Yes □No	□Yes □No
Eat leftovers	□Yes □No	□Yes □No	□Yes □No

2. Did you eat any dish containing the following foods within one week before symptoms?

Exposure	Within 1 week before symptoms	Exposure	Within 1 week before symptoms
Eat ice shavings	□Yes □No	Eat cold side dishes	□Yes □No
Eat flavored popsicle	□Yes □No	Eat pickled vegetables	□Yes □No

Eat a duck/goose	□Yes □No	Eat ginger	□Yes □No
Eat sandwiches	□Yes □No	Eat spring onion	□Yes □No
Eat hamburger	□Yes □No	Eat cold noodles	□Yes □No
Eat sushi	□Yes □No	Eat ice cream	□Yes □No

3. Did you eat any of the following fruits?

Exposure	Within 1 week before symptoms	Exposure	Within 1 week before symptoms
Strawberry	□Yes □No	Date	□Yes □No
Guava	□Yes □No	Papaya	□Yes □No
Tomato	□Yes □No	Litchi	□Yes □No
Grapes	□Yes □No	Honey peach	□Yes □No
Tangerine	□Yes □No	Sakya	□Yes □No
Orange	□Yes □No	Dragon fruit	□Yes □No
Watermelon	□Yes □No	Kiwi	□Yes □No
Apple	□Yes □No	Mango	□Yes □No
Banana	□Yes □No	Cantaloupe	□Yes □No
Pineapple	□Yes □No	Durian	□Yes □No
Pear	□Yes □No	Carrabolla	□Yes □No
Plum	□Yes □No	Grapefruit	□Yes □No
Bell apple	□Yes □No	Granadilla	□Yes □No
Cherry	□Yes □No		

4. Did you drink any of the following types of juice?

Exposure	Within 1 week before symptoms	Exposure	Within 1 week before symptoms
Lemon juice	□Yes □No	Apple juice	□Yes □No
Guava juice	□Yes □No	Sugar cane juice	□Yes □No
Tomato juice	□Yes □No	Pineapple juice	□Yes □No
Grape juice	□Yes □No	Kiwi juice	□Yes □No
Grapefruit juice	□Yes □No	Mango juice	□Yes □No
Orange juice	□Yes □No	Papaya juice	□Yes □No
Watermelon juice	□Yes □No	Mixture of fruit juices	□Yes □No

5. Did you drink any of the following forms of water or milk?

Exposure	Within 1 week before symptoms	Exposure	Within 1 week before symptoms
Drink tap water	□Yes □No	Drink raw milk	□Yes □No
Drink water from a well	□Yes □No	Drink milk tea	□Yes □No
Drink bottled water	□Yes □No	Drink mineral water [non-commercially available]	□Yes □No
